# A multi-dimensional DNS domain intelligence dataset for cybersecurity research

**DOI:** 10.1016/j.dib.2025.112062

**Published:** 2025-09-13

**Authors:** Radek Hranický, Ondřej Ondryáš, Adam Horák, Petr Pouč, Kamil Jeřábek, Tomáš Ebert, Jan Polišenský

**Affiliations:** Faculty of Information Technology, Brno University of Technology, Božetěchova 1/2, 612 00 Brno, Czech Republic

**Keywords:** Domain, DNS, TLS, WHOIS, RDAP, IP, Geolocation, Malware, Phishing

## Abstract

The escalating sophistication and frequency of cyber threats require advanced solutions in cybersecurity research. Particularly, phishing and malware detection have become increasingly reliant on data-driven approaches. This paper presents a unique dataset precisely curated to bolster research in network security, focusing on the classification and analysis of internet domains. This dataset contains information for over a million internet domains with detailed labels distinguishing between phishing, malware, and benign traffic.

Our dataset is distinctive due to its comprehensive compilation of metainformation derived from multiple sources, including DNS records, TLS handshakes and certificates, WHOIS and RDAP services, IP-related data, and geolocation details. Such rich, multi-dimensional data allows for a deeper analysis and understanding of domain characteristics that are critical in identifying and categorizing cyber threats. The integration of information from diverse sources enhances the dataset's utility, providing a holistic view of each domain's footprint and its potential security implications.

The data is formatted in JSON, ensuring versatility, accessibility for researchers, and easy integration into various analytical tools and platforms, facilitating ease of use in statistical analysis, machine learning, and other computational analyses. Our dataset's extensive volume and variety surpass any known publicly available resources in this field, making it an invaluable asset for both academic and practical development and testing of cybersecurity solutions.

This paper thoroughly describes the value of the data, details the comprehensive methodology employed in the collection process, and provides a clear description of the data structure. Such documentation is crucial for ensuring that the dataset can be effectively utilized and reapplied in a variety of research contexts. Its structured format and the broad range of included features are critical for developing robust cybersecurity solutions and can be adapted for emerging threats.

Specifications Table

**Table:** 

Subject	Computer Networks and Communications
Specific subject area	Cybersecurity, focusing on detecting phishing, malware, and benign domain names using multi-source data analysis.
Type of data	JSON dump + JSON Schema with field descriptions. Filtered.
Data collection	The dataset was compiled using domain name and URL lists from various sources. Benign domains came from the Cisco Umbrella's top million list and traffic from CESNET, a Czech academic network. Phishing domains were sourced from OpenPhish and PhishTank, while malware domains originated from platforms like URLHaus and ThreatFox. Domains were filtered to prevent mislabeling and validated via VirusTotal to eliminate false positives. Data enrichment involved DNS, TLS, RDAP/WHOIS, IP geolocation, and round-trip time measurements, using a custom Python program with MongoDB for storage. The JSON format ensured consistent structuring across the dataset.
Data source location	Data sources were various Internet servers worldwide, including those in a real ISP network of the CESNET association. The collection point was a server at Brno University of Technology, Czechia.
Data accessibility	Repository name: A Dataset of Information (DNS, IP, WHOIS/RDAP, TLS, GeoIP) for a Large Corpus of Benign, Phishing, and Malware Domain Names 2024 Data identification number: 10.5281/zenodo.13330073 Direct URL to data: https://zenodo.org/records/13330073
Related research article	R. Hranický, A. Horák, J. Polišenský, O. Ondryáš, K. Jeřábek, and O. Ryšavý “Spotting the Hook: Leveraging Domain Data for Advanced Phishing Detection”, in Proceedings of the 20th International Conference on Network and Service Management (CNSM). IEEE, 2024, pp. 1–7

## 1 Value of the Data

• The dataset provides comprehensive information about phishing, malware, and benign domains enriched with diverse metadata collected from various sources such as DNS, GeoIP, TLS, and RDAP/WHOIS. This makes it a valuable resource for all researchers in the field of network security, malware analysis, and phishing detection.
• Researchers may use the dataset to explore domain usage trends, attacker infrastructure patterns, and the lifecycle of malicious domains. Its precise labeling and multi-source structure enable statistical and comparative analysis of structural, temporal, and geographic characteristics across domain classes. The data supports detailed investigation of TTL values, certificate chains, registration details, and IP distributions. It also allows for studying cross-source feature correlations, designing data fusion strategies, and selecting robust indicators of malicious behavior.
• The dataset is also well suited for developing cybersecurity solutions that detect and mitigate domain-based threats. Such software includes intrusion detection systems, domain reputation scoring tools, and threat intelligence enrichment platforms. The detailed metadata supports building tools for anomaly detection, real-time phishing and malware classification, and proactive threat identification.
• Moreover, since the datasets are provided in the JSON format, covering raw information taken from multiple publicly available sources, it is useful for studying relationships between different data sources.
• Researchers and data scientists can use the data to train and benchmark machine learning models for the popular and important network security task of distinguishing benign and malicious network resources. We have previously shown the feasibility of using similarly shaped data for detecting phishing domain names [1,2].
• This dataset considerably extends the data used in [1] and [2], carefully curates the existing entries, adds over 96,000 new phishing entries, and introduces two entirely new subsets of benign and malware domains. We are unaware of any other dataset with such a high number of labeled domains enriched by multiple related domain information covering verified phishing and malware domains publicly available to the community.

## 2 Background

The Domain Name System (DNS) is an essential component of Internet communication, serving as the primary entry point that directs users to their intended destinations. Consequently, numerous network security solutions work with domain names to prevent users from accessing harmful endpoints. Many research teams focus on the DNS data and utilize machine learning to rank the maliciousness of each domain. Existing studies examine various domain-related features, with some [[3], [4], [5]] relying solely on data from the DNS system, while others [[6], [7], [8], [9], [10]] also incorporate IP, TLS, WHOIS/RDAP, or geolocation information. Unfortunately, many studies do not make their datasets publicly available, and those that do are often constrained by low amounts of malicious domain samples [10], or are limited in their related information provided [3], mostly to a single data source only. To address these gaps, we extend the methodologies of the existing works by collecting data on a larger set of domains and integrating several different categories of publicly available domain-related information.

## 3 Data Description

This article describes a dataset of labelled benign, phishing, and malware domain names, filtered through VirusTotal, and enriched with extensive data from various external sources. The dataset was published in the Zenodo data repository [11]. It is organized into a JSON Schema file (*schema.json*) and four JSON files, one for each of the included subsets (labels):

• *benign_umbrella.json* contains data objects for 368,956 domain names labeled as benign; the list of source domain names was based on Cisco Umbrella^1^ data.
• *benign_cesnet.json* contains data objects for 461,338 domain names labeled as benign; the list of source domain names was based on traffic in the CESNET^2^ academic network.
• *phishing.json* contains data objects for 164,425 domain names labeled as phishing; the list of source domain names was based on phishing URLs published in PhishTank^3^ and OpenPhish.^4^
• *malware.json* contains data objects for 100,809 domain names labeled as malware, the list of source domain names was based on URLs and domain names published in ThreatFox,^5^ The Firebog,^6^ Steven Black’s hostfiles^7^ consolidated from various sources, the Spam404 list,^8^ the Rescure^9^ Malicious Domain Blacklist, URLhaus^10^ and various other malicious domain name blacklists from GitHub.

The JSON files follow the MongoDB Extended JSON (v2) format^11^ in the Relaxed Mode. They contain a single top-level JSON array with the data objects represented as structured JSON objects. Each data object adheres to the included JSON Schema. Table 1 gives an overview of the schemas.

**Table 1:** A description of the data structure.

Field name	Field type	Nullable	Description
domain_name	String	No	The evaluated domain name
url	String	No	The source URL for the domain name
evaluated_on	Date	No	Date of last collection attempt
source	String	No	An identifier of the source
sourced_on	Date	No	Date of ingestion of the domain name
dns	Object	Yes	Data from DNS scan
rdap	Object	Yes	Data from RDAP or WHOIS
tls	Object	Yes	Data from TLS handshake
remarks	Object	No	DNS/RDAP/TLS evaluation dates and times
ip_data	Array of Objects	Yes	Array of data objects capturing the IP addresses related to the domain name
malware_type	String	No	The malware type/family or “unknown” (only present in *malware.json*)
DNS data (*dns* field)			
A	Array of Strings	No	Array of IPv4 addresses
AAAA	Array of Strings	No	Array of IPv6 addresses
TXT	Array of Strings	No	Array of raw TXT values
CNAME	Object	No	The CNAME target and related IPs
MX	Array of Objects	No	Array of objects with the MX target hostname, priority and related IPs
NS	Array of Objects	No	Array of objects with the NS target hostname and related IPs
SOA	Object	No	All the SOA fields, present if found at the target domain name
zone_SOA	Object	No	The SOA fields of the target’s zone (closest point of delegation), present if found and not a record in the target domain directly
dnssec	Object	No	Flags describing the DNSSEC validation result for each record type
ttls	Object	No	The TTL values for each record type
remarks	Object	No	The zone domain name and DNSSEC flags
RDAP domain data (*rdap* field)			
copyright_notice	String	No	RDAP/WHOIS data usage copyright notice
dnssec	Bool	No	DNSSEC presence flag
entitites	Object	No	An object with various arrays representing the found related entity types (e.g. *abuse, admin, registrant*). The arrays contain objects describing the individual entities.
expiration_date	Date	Yes	The current date of expiration
handle	String	No	RDAP handle
last_changed_date	Date	Yes	The date when the domain was last changed
name	String	No	The target domain name for which the data in this object are stored
nameservers	Array of Strings	No	Nameserver hostnames provided by RDAP or WHOIS
registration_date	Date	Yes	First registration date
status	Array of Strings	No	The state of the registered object (see RFC 7483, section 10.2.2)
terms_of_service_url	String	No	URL of the RDAP usage ToS
url	String	No	URL of the RDAP entity
whois_server	String	No	WHOIS server address
TLS data (*tls* field)			
cipher	String	No	TLS cipher suite description according to IANA^1^
protocol	String	No	One of “TLS”, ”TLSv1.2″, ”TLSv1.3″
certificates	Array of Objects	No	Array of objects representing the certificate chain, the first element is the root certificate
IP data (elements in the *ip_data* array)			
ip	String	No	The IP address
from_record	String	No	The type of the DNS record the address was captured from
remarks	Object	No	Ping round-trip time, “is alive” flag and RDAP/geo/asn evaluation dates and times
rdap	Object	Yes	RDAP data, similar to DNS RDAP, see the JSON Schema for details
geo	Object	Yes	Geolocation data from the GeoLite2 City database (e.g. latitude, longitude, city, country, etc.)
asn	Object	Yes	Autonomous system data from the GeoLite2 ASN database (ASN, organization, network)

1 IANA TLS Cipher Suites: https://www.iana.org/assignments/tls-parameters/tls-parameters.xhtml#tls-parameters-4.

**Fig. 1:**
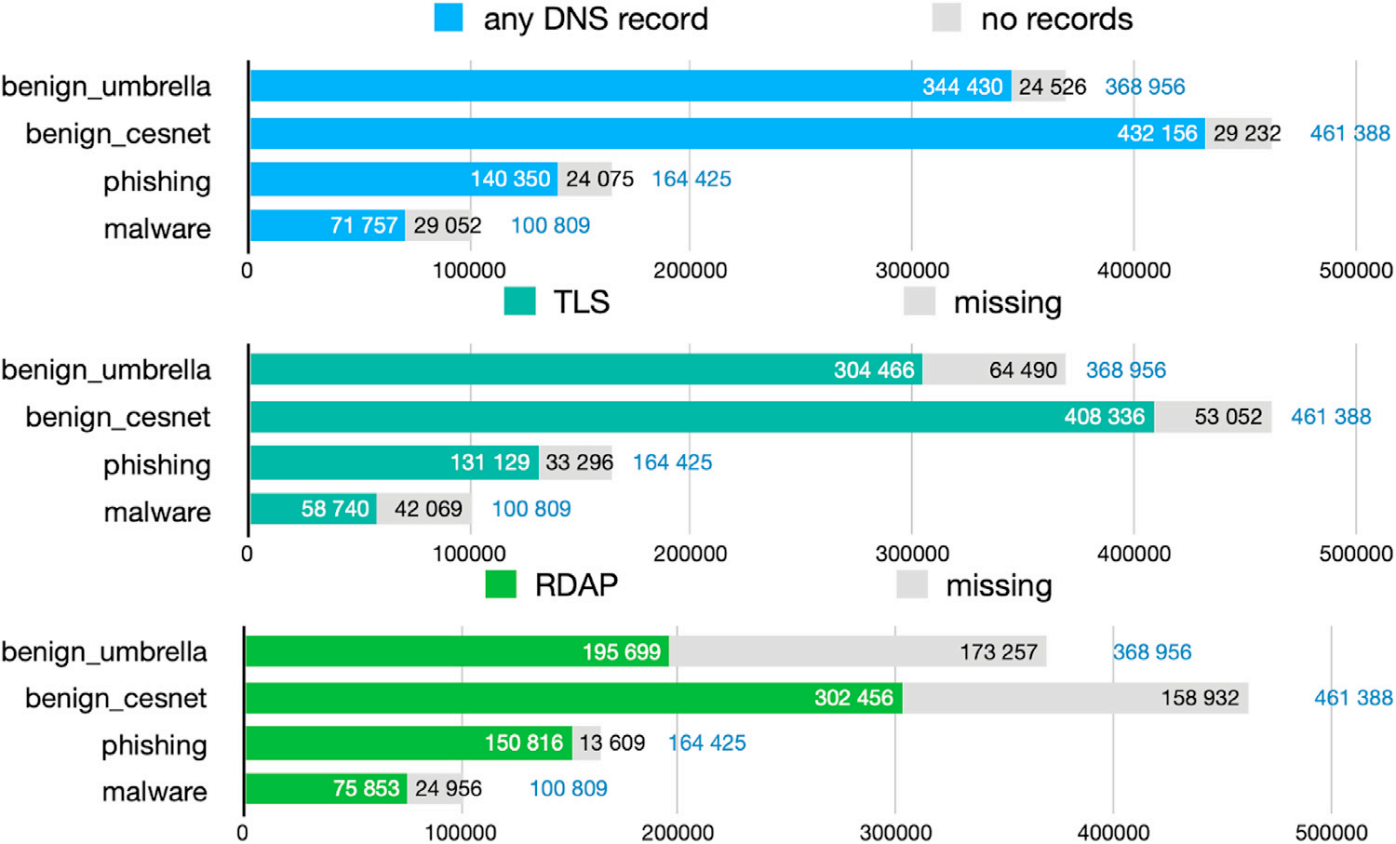
Availability of specific domain-related data types across the datasets.

**Fig. 2:**
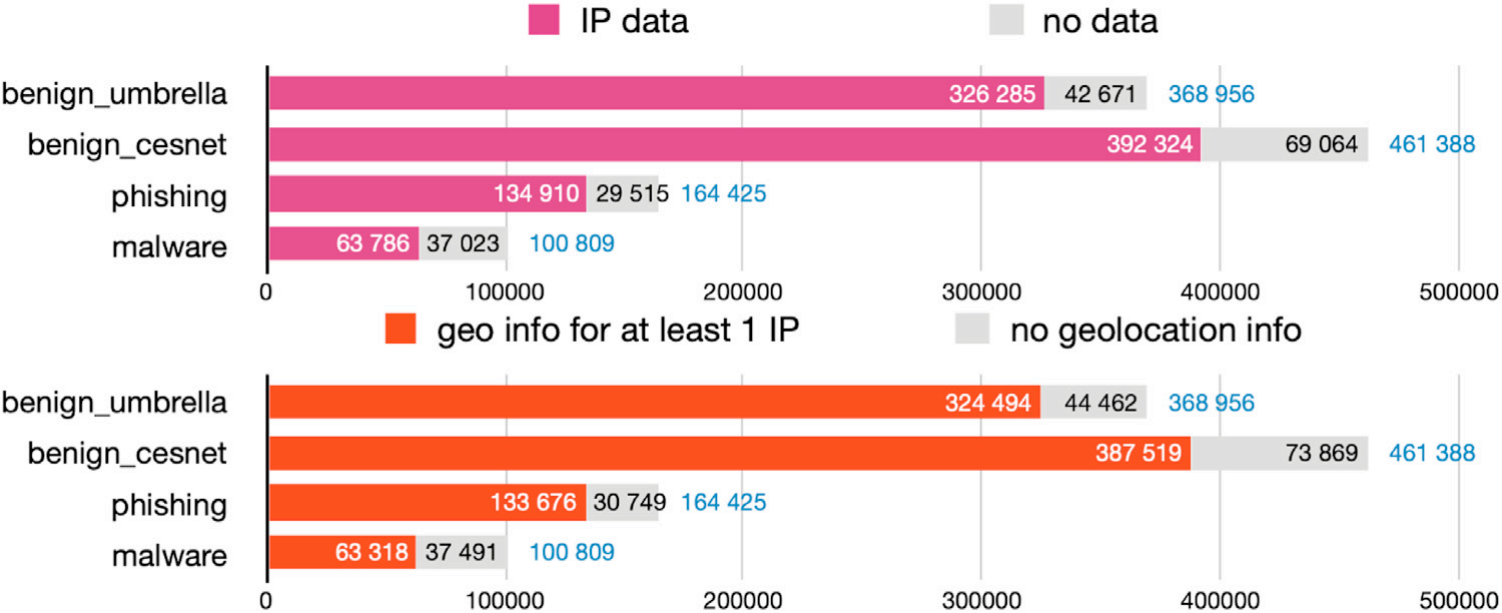
Availability of IP addresses and related data across the datasets.

Each data object captures the data collected for a domain name from DNS, RDAP or WHOIS, and from a TLS handshake; additionally, each contains an array of related IP addresses. For each IP address, the dataset includes data collected from RDAP, MaxMind’s GeoLite2 City and ASN databases,^12^ the NERD^13^ reputation system, and a round-trip time measurement originating from a single machine in the CESNET network. The number of present data types differs for each domain name. The charts in Fig. 1 show for how many data objects the DNS, TLS, and RDAP/WHOIS data collection succeeded. Similarly, Fig. 2 shows how many data objects contain at least one IP address record and at least one such record with geolocation data available. The source file for the graphs in Fig. 1, Fig. 2 is available in the source code repository [12] under the *graph_sources* directory.

A portion of the dataset is based on the datasets that we originally collected for use in [1] (between March and July 2023) and [2] (extending to November 2023). These previous datasets both consist of only two subsets, benign and phishing. In both papers, we used the same benign dataset which was a superset of *benign_umbrella*. This version described in this paper underwent additional filtering (see Section “Filtering the Dataset”) to ensure the benignity of its entries. The previous phishing datasets were both strict subsets of the *phishing* dataset described in the paper. In [1], it contained 36,993 entries (unfiltered), and in [2], it contained 68,535 entries (after a similar filtering process to the one described below). The current phishing dataset thus introduces another 96,072 entries (collected up to July 2024) that were also subject to the filtering process. Most notably, the *benign_cesnet* and *malware* datasets are new contributions (of 461,338 and 100,809 entries each) that have not been published previously. Another minor contribution in this paper is the inclusion of the JSON Schema with field descriptions.

## 4 Experimental Design, Materials and Methods

The four subsets were collected using a common process consisting of making a list of input domain names, filtering, and collecting the enrichment data. However, the first two phases differed for each subset. In this section, we first explain how we acquired the labelled domain names in each category. We show the filtering process, and finally, we describe the external data collection tool. The source code of all the custom software was published in a separate GitHub repository [12].

### 4.1 The benign domain list based on Cisco Umbrella

To acquire a set of benign domains for the dataset, we chose the public Top One Million list provided by the Cisco Umbrella platform. This selection was based on the platform's collection methodology, which utilizes the DNS resolutions of millions of users across >150 countries worldwide. The platform also includes subdomains and extends beyond domain hosting websites, covering generally popular domains regardless of the services they provide. This characteristic aligns the resulting dataset with a reliable source that mimics later real-world input for domain classifiers.

To guarantee the inclusion of only benign domains within the dataset, we performed recurrence filtering, as described by Rahbarinia et al. [13] (see *cisco_umrella_benign_load.py* in the source code repository [12]). It works by selecting only those domains that consistently appeared in the top list every month within a year's worth of archives. This process resulted in a compiled list containing 432,572 benign domains.

### 4.2 The benign domain list based on CESNET traffic

The second benign dataset was based on real-traffic domain names from a Czech academic network operated by CESNET.^14^ The CESNET domains represent a sample of domains used on the Internet regardless of their popularity or age. The domains are extracted by network monitoring probes placed in the CESNET network. The probes are configured to extract other data such as TLS SNI information in addition to traditional flow statistics. The input domain list was compiled from the TLS SNIs captured in the network.

Since data collected from the CESNET network is likely to contain sensitive information like hostnames of concrete computers in offices, labs, students' dormitories, etc., we applied an anonymization process as follows:

• For all CESNET association members,^15^ we made a list of used 2nd and 3rd-level domain names. Those are typically used for hosting web servers, mail servers, and other public services. The institutions mainly use 2nd level domains, while their departments and faculties utilize corresponding 3rd level domains. Including those in the dataset is desired.
• We removed all lower-level domain names that had a CESNET member domain suffix. An exception was made for “www” as the 4th-level domain. For instance, member.cz, department.member.cz, and www.department.member.cz were included in the dataset, while pcfrank.department.member.cz was removed.
• We also removed all domain names that contain concrete IP addresses, e.g. 1.2.3.4.something.com or 1-2-3-4.site.org.

To ensure the benignity of the domain names, we applied several filtering processes described below in Section “Filtering the domain lists”.

### 4.3 The phishing domain list

The domains for the phishing dataset were collected from OpenPhish and PhishTank. Both services publish new entries through a regularly updated feed (see OpenPhish Community Feed^16^ and PhishTank Developer Information^17^). We used a custom local instance of MISP^18^ to continuously ingest new entries from the feeds. Twice every day, we transferred the newly seen domain names from MISP to a MongoDB database through the Enrichment Data Collector and executed the collection process (see Section “Enrichment data collection”).

### 4.4 The malware domain list

**Fig. 3:**
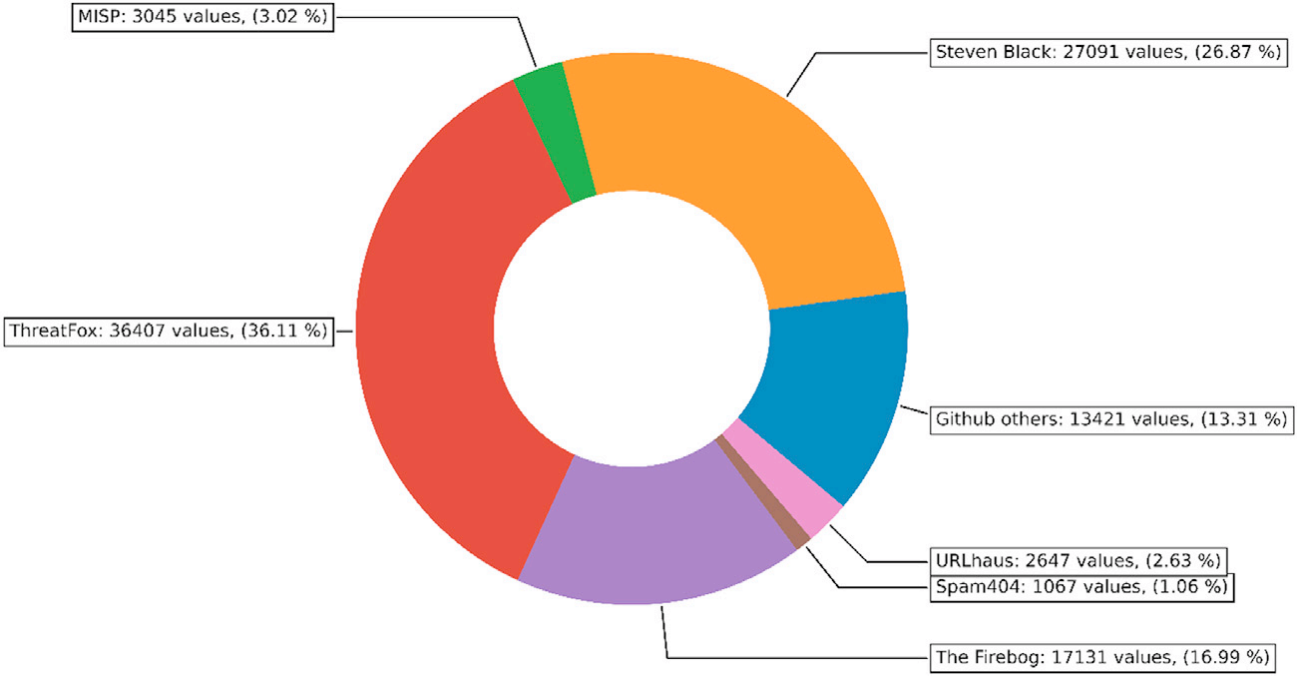
The distribution of domain name sources in the malware subset.

Malware data was collected periodically from several sources. Daily contributors were URLHaus, ThreatFox, and Rescure, where it was possible to get up to 500 domains per day from each. Steven Black’s list was also checked periodically, although it was not updated every day. The other sources were only ingested once, as they are not updated regularly. Fig. 3 shows the share of individual contributors to the malware data subset.

The one-off contributors offer text files that were loaded directly into the database using the Enrichment Data Collector. The others needed individual processing approaches. The *malware_data* directory in the source code repository [12] contains the scripts used for parsing, storing, and loading the domain names into the database. The main script *loader.py* performs the domain source collection process for all the sources except ThreatFox, which is handled in *threatfox.py*.

**Fig. 4:**
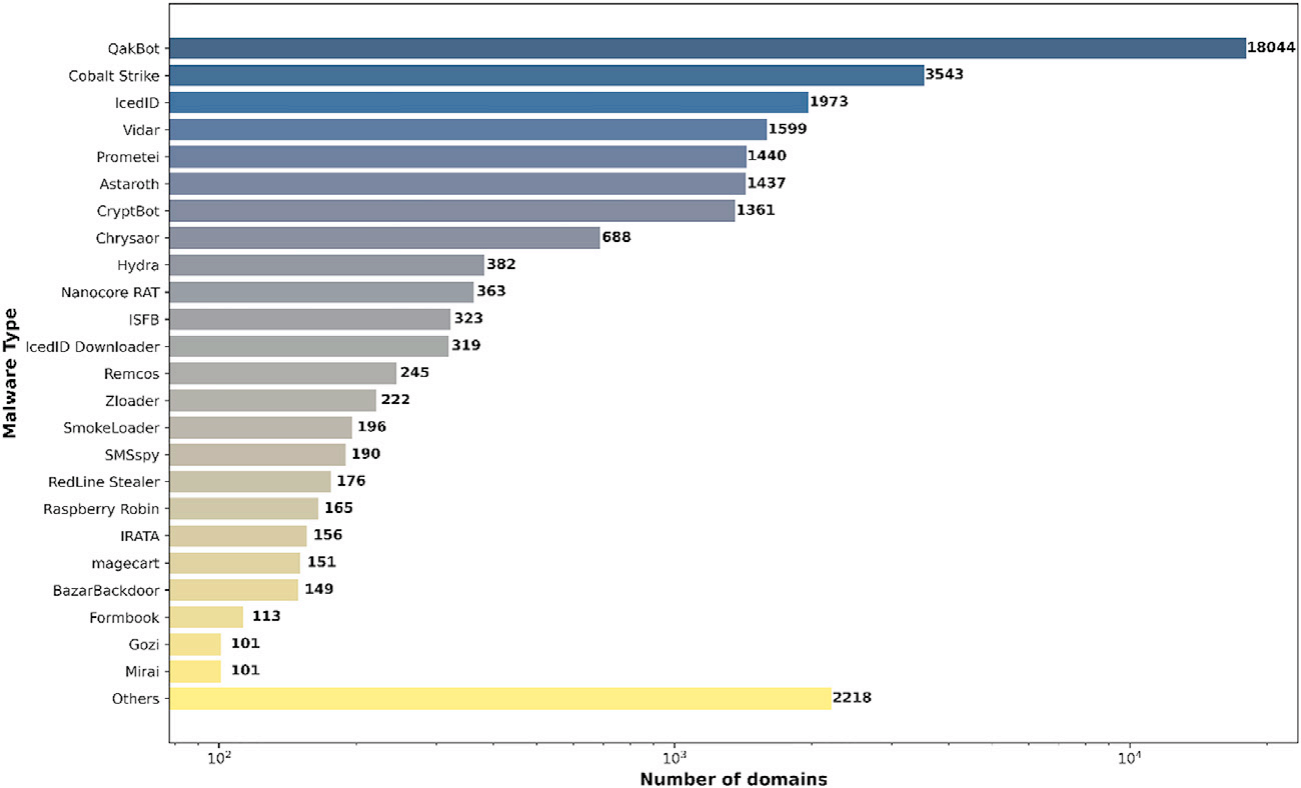
The distribution of malware types in the dataset. The X axis is logarithmic.

Some sources offered additional labels indicating the malware type or family. For malware domain names with this information available, Fig. 4 shows the share of individual malware types.

Scripts for generating the graphs in Fig. 3, Fig. 4 are available in the source code repository [12] under the *graph_sources* directory.

### 4.5 Filtering the dataset

As previously noted, our datasets incorporate domain names from diverse sources. Although the sources themselves indicate the benign, phishing, or malware character of the domains, they might have been displaced in some cases. Therefore, we chose to verify all the domain name lists via VirusTotal^19^ (VT), a renowned cybersecurity platform that can verify a URL or domain name legitimacy based on information provided by 96 security vendors. This additional verification was crucial for eliminating potential false positives and confirming the nature of the domains, resulting in a more reliable dataset.

**Fig. 5:**
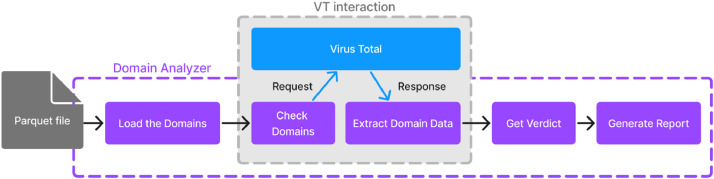
The filtering process based on VirusTotal.

The architecture outlined in Fig. 5 describes the framework for domain validation, leveraging the VT API provided to us for academic purposes. The input of the verification pipeline is a dataset of domain names and related information in the form of an Apache Parquet file. Domains are then verified by fetching data from VT and applying a decision strategy. We focus on the results of analyses provided by security vendors. Each VT report contains the number of vendors that have flagged the domain name as undetected, harmless, suspicious, or malicious. To classify a domain name as benign, i.e., ensuring it is not filtered out from our benign dataset, we required that *no* vendors had flagged it as suspicious or malicious. When filtering the phishing and malware datasets, we instead required that at least *three* vendors had flagged the domain name as suspicious or malicious.

This procedure was applied to the *benign_umbrella, phishing*, and *malware* subsets.

The code that provides this domain verification process can be found in the *data_verification* directory in the source code repository [12]. It includes all verification scripts, instructions, and example input/output.

For the *benign_cesnet* subset, a different approach, still utilizing VT, was applied due to its nature and the enormous number of records. The related code is available in the *cesnet_data* directory in the source code repository.

**Fig. 6:**
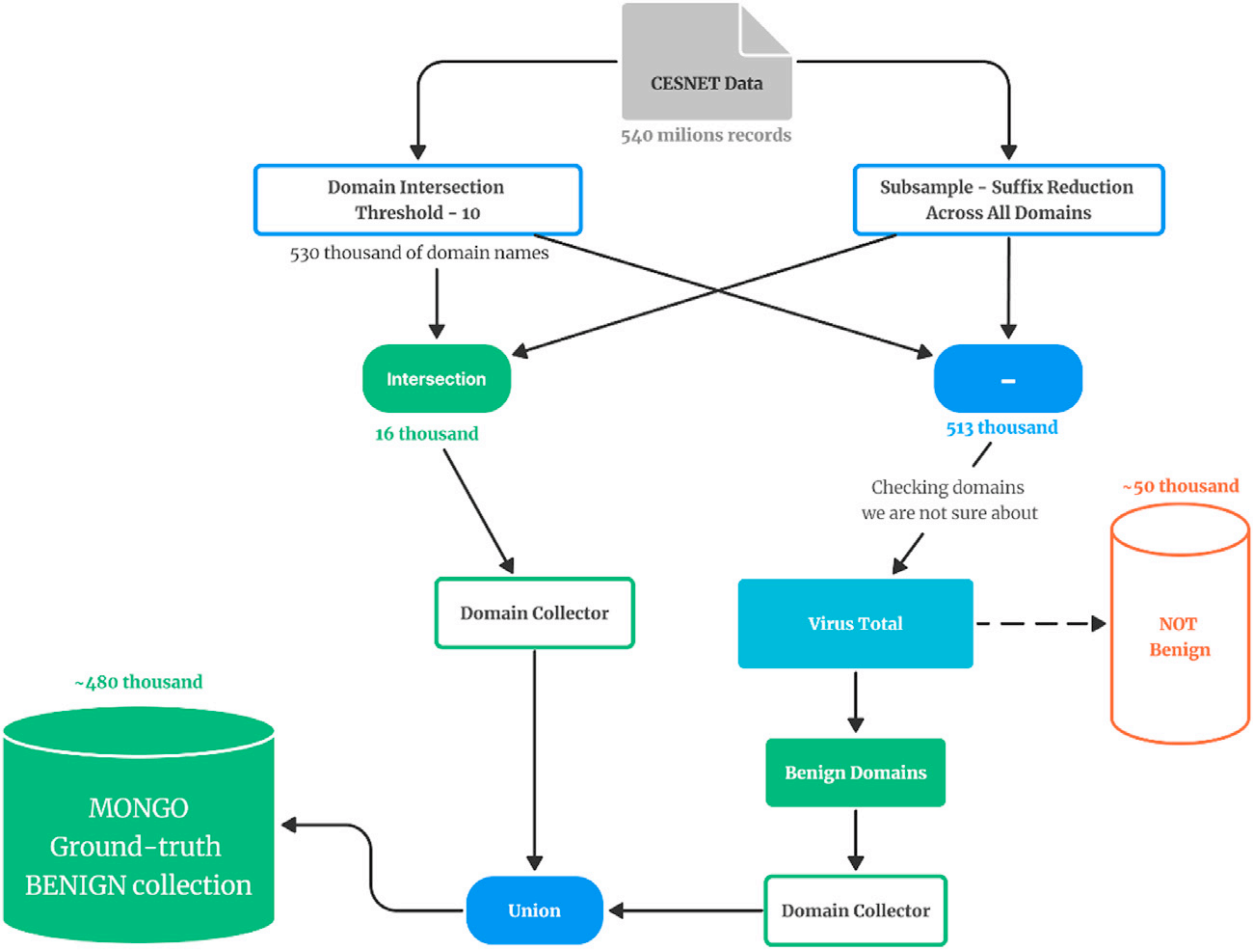
The filtration process for the domains sourced from CESNET.

The process applied to CESNET-originated domains is depicted in Fig. 6. The input set of anonymized CESNET domain names was created by collecting data periodically each month, forming the CESNET set. This set then underwent two independent filtering processes. The first one was, again, inspired by the work of Rahbarinia et al. [13]. It included a threshold-based filtering (see the *threshold_filter.py* script) that restricted the set to domain names that had been seen at least ten times across the data. Then, we further reduced the set to only the domain names that had appeared at least once each month (see the *cesnet_common_domains.sh* script). These domains were more likely to be benign due to their consistent usage.

Simultaneously, we carried out suffix reduction using the *suffix_reduction.ipynb* notebook. This process removed duplicate and closely similar domains across the dataset, refining the data by focusing on distinct domain entries. First, it grouped the inputs by a “registered domain” (that is, the domain name one level above the public suffix). In the top 50 groups by number of (sub)domains, it randomly dropped 90 %. Finally, it took a random sample of 1 million records.

Around 16,000 domains appeared in the results of both filtration processes. We considered this intersection benign without further checking. The rest of the domain names yielded by the first process that were not found in this intersection were then made subject to the same VT-based verification process as described above.

### 4.6 Enrichment data collection

The data from external sources were collected using a custom Enrichment Data Collector (EDC) program implemented in Python 3.9. The source code is included in the *collector* directory in the source code repository [12]. The *README.md* file contains detailed instructions on its usage. The program uses MongoDB for data storage.

The EDC is used in two phases: First, the domain lists must be loaded into the database using the “load” mode. This creates a stub record in the selected MongoDB collection for each input domain name. Several source formats are supported, and the EDC can also ingest domain names from MISP feeds. Then, the EDC can be started in the “resolving” mode, where it iterates through the records in the selected collection and attempts to obtain the enrichment data from external sources. Each time the EDC is executed in the resolving mode, it performs an incremental update: for each domain, it queries only those external sources queried from which data are missing. The EDC is designed modularly: the collection is handled using “resolvers”, independent Python modules that accept a domain name and return a structure with the acquired data.

The EDC collects data in a best-effort way. Timeouts in the range of seconds are used to ensure that the collection process does not stall. For all our datasets, the collection process was re-executed many times to minimize the amount of missing data, but it is not guaranteed that all data were collected for each entry. Collection attempts for various sources could have been made at different times, so a data entry may not capture the domain name at a “single” point in time.

Note that the approach to when the data was first collected differed across the input sets. Both benign sets were assembled based on past traffic spanning over a long period of time. The enrichment data were then collected in several attempts over a course of several weeks. Conversely, the phishing and malware were built by combining static sets of domains deemed malicious in the past and domain names ingested from dynamic sources (such as PhishTank) close to when they had been reported. However, malicious domains are generally shorter-lived. Thus, we executed the ingestion and collection process periodically (twice a day) to create the most complete snapshot of newly seen domains while they were still alive.

## Limitations

The dataset has limitations due to the diverse nature of the input domain lists and enrichment data sources. Benign data from Cisco Umbrella were prefiltered, containing only domains frequently observed over a year, introducing a potential bias toward long-established domains. The other benign source, CESNET, gathered data from TLS SNI fields, limiting coverage to TLS-enabled services. The users of the dataset should also adjust their analysis to mitigate the time shift bias caused by the varying data collection dates and times among the records.

Both phishing and some malware sources report full URLs, but we used only the domain part. Hosting a malicious resource, such as a phishing page or malicious code, does not necessarily imply the entire domain was created for malicious purposes; it could be a legitimate domain with a compromised subpage. To mitigate domain mislabeling, we applied VirusTotal filtering. However, some mislabeled domain names may still be present in the dataset.

Finally, collecting registration data was constrained by availability and source rate limiting. RDAP access is mandated only for gTLDs, not ccTLDs. When RDAP was unavailable, WHOIS was used instead. However, due to provider unavailability or rate limiting, some registration data may be incomplete.

## Ethics Statement

The authors affirm that they have adhered to the ethical requirements for publication in Data in Brief. The current work does not involve human subjects, animal experiments, or any data collected from social media platforms.

We explicitly confirm that no personal data about individuals was collected during this process. The data, including hostnames, IP addresses, and geolocation information, were obtained from publicly available resources such as public RDAP servers and the public GeoLite2 database. Collected data pertain solely to servers hosting Internet services and not to individuals. Furthermore, we ensured that the dataset excludes any hostnames and IP addresses associated with non-server computers, particularly those belonging to specific individuals within the CESNET group.

The authors are committed to upholding the highest ethical standards in research and publication and confirm that the data collection and analysis processes were conducted with full compliance with ethical guidelines and without compromising the privacy or personal information of individuals.

## CRediT Author Statement

**Radek Hranický:** Conceptualization, Methodology, Resources, Writing - Original Draft, Supervision, Project administration, Funding Acquisition, **Ondřej Ondryáš:** Software, Investigation, Data Curation, Formal analysis, Writing - Original Draft, **Adam Horák:** Conceptualization, Methodology, Software, Investigation, Writing - Original Draft, Visualization, **Petr Pouč:** Software, Validation, Writing - Original Draft, **Kamil Jeřábek:** Conceptualization, Software, Investigation, Data Curation, Writing – Review & Editing, **Tomáš Ebert:** Software, Investigation, Visualization, Writing - Original Draft, **Jan Polišenský:** Software, Data Curation.

## Data Availability

ZenodoA Dataset of Information (DNS, IP, WHOIS/RDAP, TLS, GeoIP) for a Large Corpus of Benign, Phishing, and Malware Domain Names 2024 (Original data).

## Footnotes

1 Cisco Umbrella Popularity List: https://s3-us-west-1.amazonaws.com/umbrella-static/index.html.

2 CESNET: https://cesnet.cz/en/.

3 PhishTank: https://phishtank.org/.

4 OpenPhish: https://openphish.com/.

5 ThreatFox: https://threatfox.abuse.ch/.

6 The Firebog: https://firebog.net/.

7 Steven Black’s consolidated hostfiles: https://github.com/StevenBlack/hosts.

8 Spam404 lists: https://github.com/Spam404/lists/.

9 Rescure: https://rescure.me/.

10 URLhaus: https://urlhaus.abuse.ch/.

15 CESNET association members: https://cesnet.cz/en/about-us/association-members.

## Footnotes

11 MongoDB Extended JSON: https://www.mongodb.com/docs/manual/reference/mongodb-extended-json/.

12 MaxMind’s GeoLite2 Free Geolocation Data: https://dev.maxmind.com/geoip/geolite2-free-geolocation-data/.

13 CESNET NERD: https://nerd.cesnet.cz/.

14 CESNET: https://cesnet.cz/en.

16 OpenPhish feeds: https://openphish.com/phishing_feeds.html.

17 PhishTank Developer Information: https://phishtank.org/developer_info.php.

18 MISP, an open-source threat intelligence and sharing platform: https://www.misp-project.org/.

19 VirusTotal: https://www.virustotal.com/.
